# Incidence and risk factors for caesarean wound infection in Lagos Nigeria

**DOI:** 10.1186/1756-0500-2-186

**Published:** 2009-09-22

**Authors:** Oliver C Ezechi, Asuquo Edet, Hakim Akinlade, Chidinma V Gab-Okafor, Ebiere Herbertson

**Affiliations:** 1 Clinical Sciences Division, Nigeria Institute of Medical Research, Lagos, Nigeria; 2 Dept. of Anaesthesia, Lagos University Teaching Hospital, Lagos, Nigeria; 3 Rao Specialist Hospital, Surulere Lagos, Nigeria

## Abstract

**Background:**

Post caesarean wound infection is not only a leading cause of prolonged hospital stay but a major cause of the widespread aversion to caesarean delivery in developing countries. In order to control and prevent post caesarean wound infection in our environment there is the need to access the relative contribution of each aetiologic factor. Though some studies in our environment have identified factors associated with post caesarean wound infection, none was specifically designed to address these issues prospectively or assess the relative contribution of each of the risk factors.

**Findings:**

Prospective multicentre study over a period of 56 months in Lagos Nigeria. All consecutive and consenting women scheduled for caesarean section and meeting the inclusion criteria were enrolled into the study. Cases were all subjects with post caesarean wound infection. Those without wound infection served as controls. Data entry and analysis were performed using EPI-Info programme version 6 and SPSS for windows version 10.0.

Eight hundred and seventeen women were enrolled into the study. Seventy six (9.3%) of these cases were complicated with wound infection. The proportion of subjects with body mass index greater than 25 was significantly higher among the subjects with wound infection (51.3%) than in the subjects without wound infection (33.9%) p = 0.011. There were also significantly higher proportions of subjects with prolonged rupture of membrane (p = 0.02), prolonged operation time (p = 0.001), anaemia (p = 0.031) and multiple vaginal examinations during labour (0.021) among the women that had wound infection compared to the women that did not have wound infection. After adjustment for confounders only prolonged rupture of membrane (OR = 4.45), prolonged operation time (OR = 2.87) and body max index > 25 (2.34) retained their association with post caesarean wound infection.

**Conclusion:**

Effort should be geared towards the prevention of prolonged rupture of fetal membrane and the reduction of prolonged operation time by the use of potent antibiotics, early intervention and use of good surgical technique. In obese women improved surgical technique and use of non absorbable sutures may suffice.

## Background

Caesarean section is a common operation in obstetric practice. The incidence is rising worldwide and the reported incidence ranges from 5 to 25% depending on the nature and area of practice [1-3].

While the operation is widely embraced and utilized in the developed world, aversion, miseries, misconception, fear, guilt and anger surround the operation in Nigeria [1,4-6]. Reasons for these includes the morbidity and mortality from the operation, prolonged hospital stay, and perceived high cost of hospital bills [1,4-6].

Earlier studies conducted by Ezechi, Fasubaa and colleagues in south western Nigeria, showed that post caesarean wound infection was not only a leading cause of prolonged hospital stay but a major cause of the widespread aversion to caesarean delivery in the region [1,7,8]. Because these women do not want relations and friends alike to know that they delivered through caesarean section, any factor that will prolong their stay in the hospital is particularly disliked and frowned at. These women have come to associate caesarean section and wound infection with long hospital stay [1,8].

Attempts to make the operation of caesarean section more acceptable to women in our environment must address these problems[1]. Post caesarean wound infection is the focus in this study.

Caesarean wound infection is a major cause of prolonged hospital stay, high hospital bills, as well as other morbidities and mortality[4,5,9]. Recovery from Caesarean section is more difficult for women who develop postoperative wound infection [10].

Rates of postoperative wound infection varied from 0 to 20.5% in a hospital survey conducted by Moir- Bussy and colleagues[11]. Two hospital based studies from Nigeria reported rates within this range[12,13].

Though the causes of caesarean wound infection are similar globally with slight regional variations, the relative contribution differ from regions to region and even from centre to centre [13]. In order to control and prevent post caesarean wound infection in our environment there is the need to access the relative contribution of each aetiologic factor.

Some studies in our environment have identified factors associated with post caesarean wound infection, however none was specifically designed to address these issues prospectively, in a multi-centre setting or address the relative contribution of each of the risk factors [11-15].

This study examined the risk factors for post caesarean wound infection prospectively in a multicentre setting using one protocol. Information obtained hopefully will be used to plan strategy to reduce post caesarean wound infection and prolonged hospital stay. Ultimately leading to lower caesarean morbidity and mortality and reduced caesarean section aversion

## Methods

The study was conducted in four multidisciplinary proprietary hospital in Lagos, Nigeria over a period of 56 months (January 2004 - August 2008). The hospitals were randomly selected from a list of hospitals in Lagos state. Informed consent and ethical clearance was obtained before commencement of the study. All consecutive and consenting women scheduled for caesarean section and meeting the inclusion criteria were enrolled into the study. Caesarean section was performed using an agreed protocol and through Pfannenstiel incision and through a transverse lower segment caesarean section. Women that had caesarean section through a midline sub umbilical vertical skin incision were excluded from analysis as per protocol. The uterine incisions were closed in two layers using chromic catgut (CCG) suture size 2, followed by CCG suture size 00 for the peritoneal layers. The rectus sheet was closed continuously using CCG suture size 1 and plain CCG suture size 00 for the apposition of subcutaneous layer. The skin was closed subcuticularly using CCG suture size 00.

The wounds were inspected on postoperative day three and wound dressings were removed and wound left open thereafter.

All subjects were interviewed on the fifth postoperative day using a standardized questionnaire by the trained research assistants. The information obtained were coded and fed into the computer using the EPI-Info programme version 6. The presence of an association between hypothesized risk factors and wound infection were tested using univariate analysis. Test of significance based on 95% confidence interval of chi square test with Yates correction were used to determine the significant variables. The significant variables were then exported to SPSS for windows statistical software package version 10.0 and by logistic regression analysis, the odds ratio was then calculated to determine the independent risk factors while controlling for confounding variables.

### Definition of terms

#### Prolonged operation time

Defined as caesarean section lasting more than one hour from skin incision to last skin stitch.

#### Post caesarean wound infection

A wound is considered infected if there were indurations and swelling of the wound edges, discharge of pus or wound dehiscence.

#### Prolonged hospital stay

Defined as hospital stay lasting more than 7 days.

#### Unbooked status

A woman who did not receive antenatal care in any of the 4 hospitals.

## Results

During the 56 months period of study, eight hundred and seventeen consecutive and consenting women scheduled for caesarean section and meeting the inclusion criteria of Pfannenstiel incision were enrolled into the study. Twenty one women that had caesarean through a midline sub umbilical incision were excluded from analysis as per protocol. Seventy six (9.3%) of the 817 cases meeting the inclusion criteria were complicated with wound infection. Seven hundred and forty one subjects without post caesarean wound infection served as controls.

Table 1 summaries the sociodemographic and obstetric characteristics of the subject in the study. The proportion of subjects with body mass index greater than 25 was significantly higher among the subjects with wound infection (51.3%) than in the subjects without wound infection (33.9%) p = 0.011. There were also significantly higher proportions of subjects with prolonged rupture of membrane (p = 0.02), prolong operation time (p = 0.001), anaemia (p = 0.031) and multiple vaginal examination during labour (0.021) among the women that had wound infection compared to the women that did not have wound infection. There was no significant difference between the cases and control in respect to maternal age, previous surgery, and prolonged labour and unbooked status.

**Table 1:** Distribution of sociodemographic and obstetric characteristics among the women.

**Variable**	**Cases** **N = 76(%)**	**Controls** **N = 741(%)**	**X^2^**	**P value**
Maternal age(years)				
• Less than 20	8(10.5)	88(11.9)		
• 20-34	61(80.3)	588(79.4)	0.13	0.93
• Greater than 35	7(9.2)	65(8.8)		
Body mass index				
• Less than 23	15(19.7)	205(27.7)		
• 23-24	22(29.0)	281(37.9)	8.97	0.0112
• Greater 25	39(51.3)	251(33.9)		
Booking status				
• Booked	57(75.0)	54273.1)		
• Unbooked	19(25.0)	199(26.9)	0.05	0.83
Prolonged rupture of membrane	11(14.5)	51(6.9)	5.66	0.02
Prolonged operation time	23(30.3)	117(19.8)	10.16	0.001
Anaemia	13(17.1)	137(18.5)	4.63	0.031
Multiple vaginal examination	29(38.2)	191(25.8)	5.36	0.021
Prolonged labour	36(47.4)	339(45.7)	0.07	0.79
Previous surgery	13(17.1)	145(19.7)	0.27	0.6

After adjustment for confounders of age, parity and unbooked status (Table 2), only three variables retained significant association with post caesarean wound infection. These variables were prolonged rupture of membrane (OR = 4.45), prolonged operation time (OR = 2.87) and body max index > 25 (2.34).

**Table 2:** Variables independently associated with caesarean wound infection.

**Variables**	**Odd ratio**	**95% confidence interval**
Prolonged rupture of membrane	4.45	2.34-8.51
Prolonged operation time	2.87	1.96-5.97
Body max index > 25	2.34	1.12-4.23

## Discussion

The incidence of post caesarean wound infection in this study of 9.3% is similar to 10% reported by Fasubaa etal [1], but much lower than that 23.4% reported by Makinde^2 ^from Ile Ife Nigeria. It is also important to state that though the rate of 9.3% is within 0 to 20.5% reported by Moir- Bussy and colleagues in a hospital survey in London, it is much higher than figures reported from most developed countries [11,12]; It could even be higher if the protocol had included cases performed through midline sub umbilical incisions. Post caesarean wound infection in this study may not be the true representative of what currently obtains in most of our secondary facilities where most of the caesarean section occurs. However, it highlights the facts that with the use of standardized protocol and good practice, it is possible to reduce cesarean wound infection from high rate of 23.4% in Ile Ife[1] to 9.3%.

While some investigators were able to demonstrate an association between maternal age, anaemia, prolonged labour, previous caesarean section, multiple vaginal examination and unbooked status and post caesarean wound infection [13-18], this study like the report of Beatle [19] could not confirm the association. This finding is not surprising in that multiple vaginal examination with sterile gloves and aseptic technique is not likely to increase infection rate. Also wound infection due to anaemia is likely to occur in severe anaemic conditions and not in mild/borderline cases in which category the majority of our patients are. Meticulous closure of potential spaces and good haemostatic technique may have reduced the incidence of haematoma collection leading to wound infection in our patients who have had previous caesarean section.

The role of prolonged rupture of membrane as a predisposing factor to developing wound infection reported by Bariweni^13^, Litta^19 ^and Okonofua[20] was confirmed in this study (p = 0.02). The risk of developing wound infection was increased by more than four times in patients having prolonged rupture of membrane when adjustment was made for the potential confounders (OR = 4.45: CI. 2.34-8.51). Normally in pregnancy, cervical mucus plug, fetal membranes and amniotic fluid all serve as barriers to infection. However when fetal membranes are ruptured, this protective effect is gradually lost with time. Bacteria are now able to transverse the cervical canal into the amniotic cavity leading to chorioamnonitis and its sequel.

That the risk of developing postoperative wound infection is considerably reduced when the operation time is short[12] was confirmed by this study (OR = 2.87; CI = 1.96-5.97). In the course of prolonged operation, there is significant tissue devitalisation resulting from tissue handling and reduced tissue perfusion.

This study also confirmed reports of several investigators that there exist a direct correlation between increasing maternal weight and higher rate of wound infection[10,21,22]. The mean body mass index of the women with wound infection was significantly higher than the women without wound infection. Even after adjustment for confounders BMI > 25 still retain significant association with wound infection (OR = 2.34, CI = 1.12-4.23). Also noted in this study is that the mean body mass index among the women with wound infection was above the normal range.

Though this study addressed some specific gaps, it still has some limitations that may pose a challenge to the generalisability of the results. The exclusions of cases of cesarean section performed through midline sub umbilical incisions from the protocol may have led to a lower wound infection rate and also excluded a potential cause of caserean wound infection and complications[14]. It is thus reasonable to avoid midline sub umbilical incision as it a major contributor to post operative morbidity, except where it is practically impossible[7].

Secondly as the study was done in a homogenous population using very standardized protocol, it may not be generalisable to a larger more heterogeneous population. However the use of patients from 4 different hospitals, chosen randomly may have ameliorated such possible effect.

Prolonged rupture of fetal membrane, prolonged operative time and BMI greater than 25 were identified as independent risk factors for caesarean wound infection in this study. They should be utilized in designing wound infection prevention and control strategies. While prolonged prophylactic antibiotics should be the standard of care in cases complicated by any of these factors. Patients with the identified risk factors presenting in secondary facilities should be referred to tertiary institution when feasible.

## Conclusion

Effort should be geared towards the prevention of prolonged rupture of fetal membrane and the reduction of prolonged operation time by the use of potent antibiotics, early intervention and use of good surgical technique. In obese women improved surgical technique and use of non absorbable sutures may suffice.

## Competing interests

The authors declare that they have no competing interests.

## Authors' contributions

OE conceived, designed and coordinated the study, data collection and as well as drafting of the manuscript. HA participated in the design of the study, data collection, and drafting the manuscript. EA participated in data collection & entry and helped to draft the manuscript. CVGO participated data collection and correction of the peer reviewed manuscript. HE participated in data collection and correction of the peer reviewed manuscript. All authors read and approved the final manuscript.
